# In Vitro Assessment of Antifungal and Antibiofilm Efficacy of Commercial Mouthwashes against *Candida albicans*

**DOI:** 10.3390/antibiotics13020117

**Published:** 2024-01-25

**Authors:** Marzena Korbecka-Paczkowska, Tomasz M. Karpiński

**Affiliations:** 1 Medi-Pharm, os. Konstytucji 3 Maja 14/2, 63-200 Jarocin, Poland; mkorbecka@wp.pl; 2 Chair and Department of Medical Microbiology, Poznań University of Medical Sciences, Rokietnicka 10, 60-806 Poznań, Poland

**Keywords:** antiseptic, yeast, treatment, oral rinse, complication

## Abstract

*Candida albicans* is the most critical fungus causing oral mycosis. Many mouthwashes contain antimicrobial substances, including antifungal agents. This study aimed to investigate the in vitro activity of 15 commercial mouthwashes against 12 strains of *C. albicans*. The minimal inhibitory concentrations (MICs), minimal fungicidal concentrations (MFCs), and anti-biofilm activity were studied. MICs were determined by the micro-dilution method using 96-well plates, and MFCs were determined by culturing MIC suspensions on Sabouraud dextrose agar. Anti-biofilm activity was evaluated using the crystal violet method. The mouthwashes containing octenidine dihydrochloride (OCT; mean MICs 0.09–0.1%), chlorhexidine digluconate (CHX; MIC 0.12%), and CHX with cetylpyridinium chloride (CPC; MIC 0.13%) exhibited the best activity against *C. albicans*. The active compound antifungal concentrations were 0.5–0.9 µg/mL for OCT products and 1.1–2.4 µg/mL for CHX rinses. For mouthwashes with CHX + CPC, concentrations were 1.56 µg/mL and 0.65 µg/mL, respectively. Products with polyaminopropyl biguanide (polyhexanide, PHMB; MIC 1.89%) or benzalkonium chloride (BAC; MIC 6.38%) also showed good anti-*Candida* action. In biofilm reduction studies, mouthwashes with OCT demonstrated the most substantial effect (47–51.1%). Products with CHX (32.1–41.7%), PHMB (38.6%), BAC (35.7%), *Scutellaria* extract (35.6%), and fluorides + essential oils (33.2%) exhibited moderate antibiofilm activity. The paper also provides an overview of the side effects of CHX, CPC, and OCT. Considering the in vitro activity against *Candida albicans*, it can be inferred that, clinically, mouthwashes containing OCT are likely to offer the highest effectiveness. Meanwhile, products containing CHX, PHMB, or BAC can be considered as promising alternatives.

## 1. Introduction

*Candida albicans* is a yeast-like fungus that naturally forms part of the microbiota in the digestive tract. In healthy individuals, the colonization of *Candida* yeasts in the oral cavity typically ranges from 35% to 80% [1]. In immunocompromised persons, *C. albicans* stands as the primary cause of both mucosal and systemic fungal infections, responsible for approximately 70% of such infections globally [1]. Invasive mycosis caused by *C. albicans* is estimated to result in over 400,000 cases annually, with a mortality rate ranging from 46% to 75% [2,3]. The development of candidosis in the oral cavity is influenced by various factors, including systemic diseases such as diabetes, leukopenia, HIV/AIDS, cancer, xerostomia, and autoimmune diseases. Poor oral hygiene, smoking, a carbohydrate-rich diet, the use of antibiotics or steroids, and immunosuppressive conditions are additional contributing factors. Other predisposing elements include age (both in newborns and older individuals), pregnancy, and nutritional deficiencies in iron, folic acid, and vitamins [1,4]. Furthermore, intraoral factors like acrylic dentures and orthodontic appliances can contribute to a higher incidence of candidosis [5,6]. Oral candidosis can manifest in various clinical forms, including pseudomembranous, chronic erythematous, angular cheilitis, and hypertrophic candidosis [7]. Research suggests that *C. albicans* plays a crucial role in the development of multipathogenic infections, including periodontitis, dentinal caries, and oral carcinoma [8,9,10].

In the oral cavity, *Candida albicans* can exist in three morphological forms—blastospores, pseudohyphae, and hyphae—and it possesses multiple virulence factors. The hyphal form produces the candidalysin enzyme, which can damage host cells, potentially contributing to systemic infection [11]. Adhesins of *C. albicans* play a crucial role in adhering to host cells by binding to ligands, such as proteins. Additionally, *C. albicans* secretes hydrolytic enzymes, including proteases, lipases, and hemolysins, enabling the invasion of mucosal surfaces and blood vessels while evading the host’s immune response [12]. Most infections caused by *C. albicans* are linked to the formation of biofilms on the surfaces of host cells or abiotic surfaces. Biofilms are characterized by high resistance to various bactericidal agents, including antifungals [13]. According to guidelines, the recommended treatment for oral candidosis includes clotrimazole, miconazole, nystatin, or fluconazole. However, due to the increasing resistance of yeasts to antimicrobial drugs, there is a growing tendency to use locally acting substances [14,15]. In the oral cavity, mouthwashes are commonly employed, often containing antiseptic substances with potential antifungal effects.

While exploring the PubMed database, it becomes apparent that the majority of publications concerning the impact of mouthwashes on *Candida* fungi concentrate on a single product or only a limited number of commercial mouthwashes [16,17,18,19,20,21]. Simultaneously, there is a scarcity of publications investigating both MIC values and antibiofilm activity. Our search yielded only three articles in the database that assessed both the efficacy of commercial mouthwashes against planktonic forms (MIC) and biofilm [16,22,23].

In this study, we present research focused on both the planktonic form and the biofilm of *C. albicans*. The simultaneous assessment of 15 commercial mouthwashes, each with distinct compositions of primary antimicrobial substances, sets this study apart. The focus on examining the efficacy against both planktonic and biofilm forms enhances our understanding of potential antifungal agents in the context of oral health, providing valuable insights for addressing the challenges posed by *C. albicans* infections and conventional treatments. The study aims to assess the in vitro antifungal efficacy of 15 commercial mouthwashes against 12 *Candida albicans* strains.

## 2. Results

### 2.1. Antimicrobial Activity (MIC/MFC)

Mouthwashes containing octenidine dihydrochloride (OCT), chlorhexidine digluconate (CHX), and a combination of CHX and cetylpyridinium chloride (CPC) demonstrated the most potent antifungal activity. MIC levels were exceptionally low (<0.5%) for the majority of them, with some MICs as low as 0.005%. The antifungal activity of mouthwashes with OCT or CHX was noted at about 1000-fold dilutions, a characteristic not observed in other tested products.

Mouthwashes containing polyhexanide (PHMB) or benzalkonium chloride (BAC) exhibited good antifungal activity, with mean MICs below 10%. A mouthwash with moderate antifungal activity contained alcohol, fluorides, and essential oils (mean MIC 16.67%). Conversely, mouthwashes with the weakest action against *C. albicans* included those with Oraflur fluoride, plant extracts, and diclofenac. In these cases, mean MIC values ranged between 31.25% and 70.83%.

The MFC/MIC ratio was the same for eleven mouthwashes and slightly higher for four products. The MIC and MFC values are presented in Table 1. Additionally, the table displays the MIC values (in µg/mL) of the primary antimicrobial components of the mouthwashes. The MIC for the antifungal drug fluconazole, serving as a control, is also provided. The MFC/MIC ratio falls between 1 and 2, signifying that all mouthwashes exhibit fungicidal activity (Table 1).

When scrutinizing the differences between OCT and CHX or CHX + CPC, no statistically significant disparities surfaced (*p* ≥ 0.05). This suggests a comparable ability to inhibit the growth of planktonic *C. albicans* for these formulations. However, when OCT was juxtaposed with mouthwashes containing PHMB, BAC, fluorides (F) + essential oils (EO), fluorides + Olaflur, plant extracts, and diclofenac, substantial differences emerged (*p* < 0.001). The obtained *p*-values indicate that, mainly, formulations with F + EO, F + Olaflur, plant extracts, and diclofenac exerted statistically weaker effects on inhibiting *C. albicans* growth than OCT. Turning attention to CHX or CHX + CPC, no significant differences were discerned when compared to PHMB (*p* ≥ 0.05). However, CHX did exhibit a statistically significant divergence (*p* < 0.05) when measured against BAC. Comparisons of CHX or CHX + CPC to mouthwashes with F + EO, F + Olaflur, plant extracts, and diclofenac demonstrated highly significant differences (*p* < 0.001), highlighting pronounced variations in antifungal efficacy. PHMB showcased comparable MICs to BAC, F + EO, and plant extracts (*p* ≥ 0.05). Nevertheless, it showed a significant difference (*p* < 0.05) compared to F + Olaflur and diclofenac, suggesting differential antifungal efficacy. Intergroup comparisons between BAC, F + EO, F + Olaflur, plant extracts, and diclofenac showed no statistically significant differences (*p* ≥ 0.05) (Table 2).

### 2.2. Antibiofilm Activity

In the study of antibiofilm activity, none of the products achieved complete biofilm destruction during the 24 h incubation period. The highest level of biofilm destruction (47% to 51.1%) was observed with octenidine mouthwashes. Eludril Classic, a product containing CHX, exhibited slightly lower activity (41.7%). Mouthwashes with PHMB, BAC, and *Scutellaria* extract demonstrated a moderate reduction (35.6% to 38.6%) in biofilm. Other mouthwashes with CHX and fluorides + essential oils removed biofilm in the range of 32.1% to 33.2%. Products containing CHX + CPC, Oraflur, or diclofenac showed the lowest antibiofilm effect (26.4% to 29.2%) (Table 3). It is important to note that the results for Dentosept were excluded from the analysis due to the inability to remove the color of this product in the biofilm, leading to a positive result. This outcome was considered likely unrelated to actual biofilm growth and was treated as a false result.

## 3. Discussion

In this study, it was demonstrated that mouthwashes containing OCT, CHX, or CHX + CPC exhibit the most effective anti-*Candida albicans* effect. The MIC values for these solutions were less than 0.5%. When calculating the concentration of the active substance in the mouthwashes, the mean MICs for OCT were 0.5–0.9 µg/mL, and for CHX they ranged from 1.1 to 2.4 µg/mL. Other studies have also highlighted the excellent antifungal effects of CHX, CPC, and OCT. In the paper by Fu et al., the results obtained for mouthwashes were similar to those obtained in this study. The MIC values for CHX were 0.78–1.56 µg/mL, and for CPC they were 0.05–1.56 µg/mL [22]. In the research conducted by Di Lodovico et al., the activity of commercial mouthwashes containing CHX at concentrations of 0.05–0.12% was evaluated. The MICs of all rinses against *C. albicans* ranged from 0.02% to 0.09% [24]. The aforementioned values for CHX and CPC are comparable to those obtained in our study.

Regarding OCT, the study by Koburger et al. yielded results comparable to ours. The values of MIC and MFC, determined according to the DIN58940-7 and 58940-82 standards, were 1 µg/mL [25]. However, it is worth noting a publication where the MIC for OCT differs significantly. In the article by Tirali et al., the MIC values of Octenisept (containing 0.1% OCT) for *C. albicans* were reported as low as 0.002 µg/mL [26].

When considering mouthwashes other than those containing OCT or CHX, Vlachojannis et al. demonstrated, similar to our findings, a moderate effect of Listerine against *Candida* with an MIC ranging between 6.25% and 12.5% [17]. In the discussion, it is pertinent to mention another publication [27] which failed to show the antifungal effect of the mouthwashes we tested: Dentosept, Eludril Classic, and Listerine Total Care. This is perplexing, particularly considering that Eludril Classic exhibits excellent activity in our results, similar to other CHX products. Furthermore, the authors indicate high activity against *C. albicans* for pure chlorhexidine, the mouthwash Corsodyl with 0.2% CHX, and Octenidol containing 0.05% OCT [28].

In the antibiofilm activity study, the most pronounced effect was observed with OCT mouthwashes. Mouthwashes with CHX, PHMB, BAC, *Scutellaria* extract, and fluorides + essential oils exhibited lower activity. Unfortunately, comparing our results with other studies is challenging due to the limited number of articles on the impact of mouthwash on *C. albicans* biofilm, the diverse methodologies employed in biofilm research, and variations in incubation times.

In the case of OCT, the eradication of *C. albicans* biofilm from fibroblast-covered cellulose carriers ranged between 70% and 80%. Unfortunately, the article does not provide exact values [29]. De Oliveira et al. [30] demonstrated a 44.5% reduction in the amount of biofilm under the influence of 0.12% CHX. This result is slightly higher, ranging from a few to a dozen percent, than the reduction observed in our study. In another paper, the average reductions in *C. albicans* biofilm were reported to be as much as 60.9% [31] and 70.6% [32]. Such a significant difference may be attributed to variations in research methodology; for example, hydroxyapatite disks were used for biofilm extraction and the quantification of viable cells.

In our studies, biofilm reduction with other mouthwashes ranged from 26.4% to 38.6%. Dudek-Wicher et al. demonstrated the most potent eradication against *C. albicans* biofilm with PHMB at 83.6% and CPC at 84.2% [32]. The eradication of *C. albicans* biofilm from fibroblast-covered cellulose carriers by PHMB was reported to be between 70% and 80%. Unfortunately, the article does not provide exact values [29]. In another study, the reduction in biofilm mass after PHMB treatment ranged from 53.5% to 57.6% [31]. The smallest removal (26.4%) of *C. albicans* biofilm was observed for diclofenac in our study. In the research by Alem and Douglas [33], biofilm reduction under the influence of diclofenac was two times higher, amounting to 57.6%. This difference may be attributed to other methodologies, such as the use of XTT and a 48 h incubation period. Due to biofilm discoloration with Dentosept, the results obtained by us were not considered for analysis. It is possible that this is why we did not find any articles on the antibiofilm effect of this mouthwash.

The treatment of oral candidiasis is typically prolonged, requiring the use of medication over several weeks [34]. The additional use of mouthwashes helps reduce the population of pathogenic microorganisms, including *Candida* species [35]. However, the use of chlorhexidine (CHX) for just two weeks may result in side effects. The most common side effects include discoloration of the teeth, tongue, and fillings. CHX mouthwash can also lead to taste disturbances, irritation of the mucous membranes, dry mouth (xerostomia), increased calculus formation, burning sensations, desquamation of the oral mucosa, parotid gland swelling, and oral paresthesia [36,37]. CHX may further cause allergic contact dermatitis, urticaria, or anaphylactic reactions. It is estimated that allergic reactions may occur in 2% of CHX users, primarily after repeated applications [38]. CHX has been demonstrated to have cytotoxic effects on human gingival fibroblasts, periodontal ligament cells, and alveolar bone cells [36]. Currently, an emerging issue related to CHX is the development of transferable resistances and cross-resistance to other substances, such as benzalkonium chloride, triclosan, and some antibiotics [39].

The most common side effects of cetylpyridinium chloride (CPC) include staining, taste alteration, and mucosal irritation [40]. Similar to CHX, CPC exhibits a high cytotoxic effect, particularly towards keratinocytes and fibroblasts [41]. In the case of octenidine dihydrochloride (OCT), the most common side effects include dysgeusia, tongue discoloration, and headaches [42]. Unlike CHX and CPC, OCT does not have genotoxic or carcinogenic effects, and it possesses a low cytotoxic potential towards host cells [15]. The biocompatibility index for OCT is >1, indicating that it has microbicidal efficacy and tolerability against mouse fibroblasts in vitro [39].

Our study has certain limitations. When selecting mouthwashes, we considered products with diverse compositions in terms of active substances that are available in Europe. Unfortunately, for many products, manufacturers do not provide the concentration of active ingredients. The absence of these data partially restricts the interpretation of the obtained results. Additionally, research and financial constraints limited our ability to test many other mouthwashes available on the market.

## 4. Materials and Methods

### 4.1. Mouthwashes

In this study, 15 commercial mouthwashes were utilized. Various products available on the European market with concentrations of primary antimicrobial compounds were selected for testing, excluding three mouthwashes containing plant substances (extracts or essential oils). The main substances and compositions of the studied rinses are presented in Table 4. The primary antimicrobial compounds included octenidine dihydrochloride (OCT), chlorhexidine digluconate (CHX), cetylpyridinium chloride (CPC), polyaminopropyl biguanide (PHMB), benzalkonium chloride (BAC), alcohol, fluorines (F), Oraflur, essential oils (EO), plant extracts, and diclofenac.

### 4.2. Fungal Strains

The tests were carried out on 10 clinical strains of *C. albicans*, all obtained from the oral cavities of individuals diagnosed with candidosis as part of routine microbiological diagnostics. The materials from the collected swab were cultured on CHROMagar *Candida* medium (Graso Biotech, Starogard Gdański, Poland). After 24 h of incubation, yeasts that grew as consistent green colonies were further identified using the biochemical test Integral System Yeasts Plus (Graso Biotech, Starogard Gdański, Poland). Additionally, reference strains of *C. albicans* ATCC 10231 and *C. albicans* ATCC 14053 (LGC Standards, Łomianki, Poland) were included. All yeast cultures were grown at 35 °C for 24 h on Sabouraud dextrose agar (Graso Biotech, Starogard Gdański, Poland).

### 4.3. Minimal Inhibitory Concentrations (MICs)

The minimal inhibitory concentrations (MICs) of the mouthwashes were determined using the micro-dilution method in 96-well plates (Nest Scientific Biotechnology, Wuxi, China). The studies were conducted following the methodology described in our previous publication [43]. In brief, 90 µL of tryptic soy broth (TSB, Graso Biotech) and 10 µL of fungal suspension were added to each well, resulting in a final inoculum concentration of 10^6^ CFU/mL. The suspension was prepared using McFarland standards. Serial dilutions of each mouthwash were carried out to obtain the following concentrations: 100%, 50%, 25%, 12.5%, 6.25%, 3.125%, 1.56%, 0.78%, 0.39%, 0.2%, and 0.1%. For mouthwashes where the MIC was determined below the lowest dilution (0.1%), additional tests were conducted with concentrations of 1.56%, 0.78%, 0.39%, 0.2%, 0.1%, 0.05%, 0.024%, 0.012%, 0.006%, 0.003%, and 0.0015%. The plates were incubated at 35 °C for 24 h. MIC was determined through visual analysis, quantifying the lowest percentage of antiseptic concentration inhibiting *C. albicans* growth. Additionally, 10 µL of a 1% aqueous solution of 2,3,5-triphenyl-tetrazolium chloride (TTC; Sigma Aldrich, Poznań, Poland) was added to each well to confirm yeast growth through a color reaction.

### 4.4. Minimal Fungicidal Concentrations (MFCs)

The minimal fungicidal concentrations (MFCs) were determined by culturing 10 µL suspensions from the MIC tests on Sabouraud dextrose agar (Graso Biotech). The MFC was defined as the lowest concentration of the mouthwash that inhibited microbial growth on the agar plate [24].

### 4.5. MFC/MIC Ratio

The MFC/MIC ratio is used as a criterion to distinguish between fungistatic and fungicidal effects. When the ratio is ≤4, the samples are considered fungicidal agents. Conversely, a ratio ≥8 indicates a fungistatic mode of action [44].

### 4.6. Anti-Biofilm Activity Test

The anti-biofilm activity was assessed using the crystal violet method [43]. Two oral *C. albicans* strains exhibiting the most robust biofilm formation (strongly adherent) were utilized in this study. The interpretation of biofilm production followed the criteria outlined by Długaszewska et al. [45] The mean optical density (OD) of the negative control served as the cut-off. All strains were categorized as follows: non-adherent (OD ≤ ODc), weakly adherent (ODc < OD ≤ 2 × ODc), moderately adherent (2 × ODc < OD ≤ 4 × ODc), or strongly adherent (OD > 4 × ODc), where ODc was the mean OD of control probes + 3 SD.

**Figure 1:**
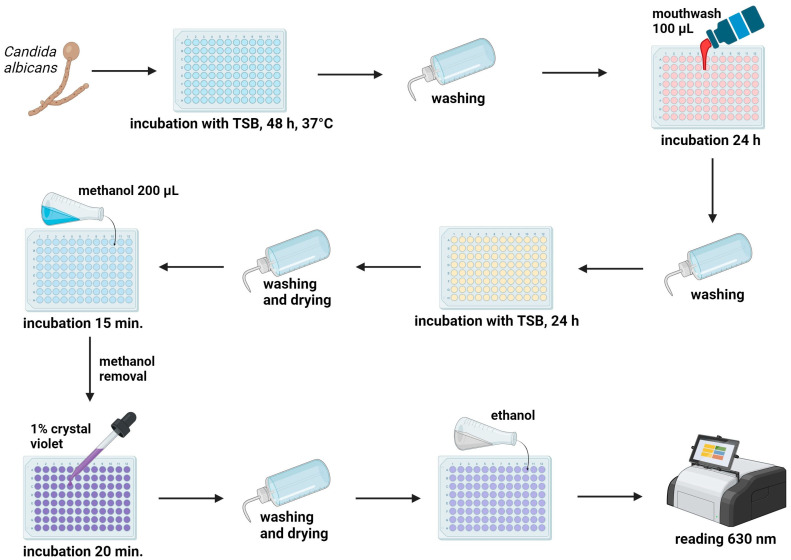
Methodology for the anti-biofilm activity test: Biofilms were developed in 96-well plates and the study was conducted in accordance with the provided figure. The optical density (OD) was assessed using a microplate reader to quantify the biofilm. Created with BioRender.com.

Both isolates of *C. albicans* were suspended to a concentration of 10^6^ CFU/mL, as per McFarland (McF) standards [46], using a densitometer (DEN-1, BioSan, Riga, Latvia). Biofilms were formed in 96-well plates with tryptic soy broth (TSB) for 48 h at 37 °C. After the incubation period, the wells were rinsed with PBS and 100 µL of mouthwash solution was added for a 24 h duration. Subsequently, the plates were washed with PBS and incubated with TSB for an additional 24 h at 37 °C. Following incubation, the wells were rinsed, dried, and fixed with 200 µL of methanol for 15 min. Next, alcohol was removed and wells were stained with a 1% crystal violet solution for 20 min. After three washes with PBS, the wells were dried, and 96% ethanol was added to dissolve the crystal violet. The optical density (OD) was measured at 630 nm using a Microplate reader 800 TS (BioTek, Waltham, USA) to quantify the biofilm (Figure 1). The tests were repeated three times for each strain. The percentage of biofilm removal was determined using the following formula:

% Biofilm growth = 100 × (Sample OD630 − Control OD630)/(Control OD630)

### 4.7. Statistics

The mean and standard deviation (SD) of the MIC and MFB values of mouthwashes against C. albicans strains were calculated. The Kruskal–Wallis test, followed by post hoc tests, were employed to assess the statistical significance of differences in the MICs of the fungi. Statistical significance was considered at the level of *p* < 0.05. The data were analyzed using InStat3 software 3.10 (GraphPad Software, Boston, MA, USA).

## 5. Conclusions

1. Among 15 commercial mouthwashes, those containing OCT, CHX, or CHX + CPC demonstrate the most effective activity (MIC, MFC) against *Candida albicans*. Products with PHMB or BAC also exhibit good antifungal action.
2. Mouthwashes containing OCT display the most potent activity against *Candida* biofilm. Products with CHX, PHMB, BAC, *Scutellaria* extract, and fluorides + essential oils show a moderate antibiofilm effect.
3. Considering the in vitro activity against *Candida albicans*, it can be inferred that, clinically, mouthwashes containing OCT are likely to offer the highest effectiveness. Meanwhile, products containing CHX, PHMB, or BAC can be considered as promising alternatives.

## Figures and Tables

**Table 1:** Mean and standard deviation (SD) values of obtained minimal inhibitory concentrations (MICs) of mouthwashes, minimal fungicidal concentrations (MFCs), MFC/MIC ratios and MIC of main antifungal compound in mouthwashes.

Mouthwash	MIC (% Concentration of Commercial Product), Mean ± SD (Range)	MFC (% Concentration of Commercial Product), Mean ± SD (Range)	MFC/MIC	MIC of Main Antifungal Compound (in µg/mL), Mean ± SD
Fluconazole—control antifungal	-	-		2.13 ± 2.23 µg/mL
Octenident	0.10 ± 0.05 (0.05–0.2)	0.10 ± 0.05 (0.05–0.2)	1	0.5 ± 0.25 µg/mL
Octenisept Oral Mono	0.09 ± 0.04 (0.05–0.2)	0.09 ± 0.04 (0.05–0.2)	1	0.9 ± 0.4 µg/mL
Eludril Classic	0.12 ± 0.05 (0.05–0.2)	0.12 ± 0.05 (0.05–0.2)	1	1.1 ± 0.5 µg/mL
Corsodyl	0.12 ± 0.09 (0.05–0.39)	0.12 ± 0.09 (0.05–0.39)	1	2.4 ± 1.8 µg/mL
SeptOralMed	0.12 ± 0.09 (0.05–0.39)	0.12 ± 0.09 (0.05–0.39)	1	2.4 ± 1.8 µg/mL
Perio Aid Intensive Care	0.13 ± 0.05 (0.1–0.2)	0.13 ± 0.05 (0.1–0.2)	1	1.56 ± 0.6 µg/mL CHX, 0.65 ± 0.25 µg/mL CPC
Gum Paroex	0.13 ± 0.06 (0.05–0.2)	0.13 ± 0.06 (0.05–0.2)	1	1.56 ± 0.6 µg/mL CHX, 0.65 ± 0.25 µg/mL CPC
ProntOral	1.89 ± 0.78 (0.78–3.125)	1.89 ± 0.78 (0.78–3.125)	1	28.35 ± 11.7 µg/mL
Fomukal	6.38 ± 3.30 (1.56–12.5)	6.51 ± 3.11 (3.125–12.5)	1–2	6.03 ± 2.23 µg/mL
Listerine Total Care	16.67 ± 7.69 (6.25–25)	18.23 ± 7.28 (6.25–25)	1–2	36.7 ± 16.9 µg/mL
Baikadent mint	31.25 ± 14.60 (12.5–50)	33.33 ± 12.31 (25–50)	1–2	the inability to calculate
Dentosept	45.83 ± 9.73 (25–50)	45.83 ± 9.73 (25–50)	1	the inability to calculate
Meridol Gum Protection	43.75 ± 11.31 (25–50)	43.75 ± 11.31 (25–50)	1	109.4 ± 28.3 µg/mL
Elmex Sensitive Plus	47.92 ± 7.22 (25–50)	47.92 ± 7.22 (25–50)	1	119.8 ± 18.1 µg/mL
Glimbax	70.83 ± 25.75 (50–100)	75.00 ± 26.11 (50–100)	1–2	524.1 ± 190.6 µg/mL

**Table 2:** Statistical analysis of minimal inhibitory concentrations (MICs) of mouthwashes based on the presence of the main antimicrobial compound.

Mouthwashes with:	OCT	CHX and CHX + CPC	PHMB	BAC	F + EO	Olaflur + F	Plant Extracts	Diclofenac
OCT	-	ns	*	**	***	***	***	***
CHX and CHX + CPC	ns	-	ns	*	***	***	***	***
PHMB	*	ns	-	ns	ns	*	ns	*
BAC	**	*	ns	-	ns	ns	ns	ns
F + EO	***	***	ns	ns	-	ns	ns	ns
Olaflur + F	***	***	*	ns	ns	-	ns	ns
Plant extracts	***	***	ns	ns	ns	ns	-	ns
Diclofenac	***	***	*	ns	ns	ns	ns	-

ns—no difference, *p* ≥ 0.05; * means *p* < 0.05; ** means *p* < 0.01; *** means *p* < 0.001. OCT—octenidine dihydrochloride; CHX—chlorhexidine digluconate; CPC—cetylpyridinium chloride; PHMB—polyaminopropyl biguanide (polyhexanide); BAC—benzalkonium chloride; F—fluorides; EO—essential oils.

**Table 3:** Mean and standard deviation (SD) values of *Candida albicans* biofilm growth reduction after 24 h of incubation with the studied mouthwashes.

Mouthwash	*C. albicans* Biofilm Reduction, Mean ± SD
Octenident	47.0 ± 10.5
Octenisept Oral Mono	51.1 ± 13.1
Eludril Classic	41.7 ± 5.1
Corsodyl	32.6 ± 5.4
SeptOralMed	32.1 ± 4.8
Perio Aid Intensive Care	29.2 ± 5.2
Gum Paroex	27.6 ± 5.6
ProntOral	38.6 ± 20.0
Fomukal	35.7 ± 13.0
Listerine Total Care	33.2 ± 22.2
Baikadent mint	35.6 ± 20.8
Dentosept	Rejected due to coloration of the biofilm
Meridol Gum Protection	27.2 ± 20.1
Elmex Sensitive Plus	28.4 ± 16.5
Glimbax	26.4 ± 10.9

**Table 4:** The composition of 15 commercial mouthwashes used in this study.

Mouthwash (Producer)	Main Antimicrobial Components	Other Components
Octenident^®^ (Schülke & Mayr GmbH, Norderstedt, Germany)	Octenidine HCl (OCT; 500 µg/mL)	Aqua, PEG-40 hydrogenated castor oil, glycerin, aroma, sodium gluconate, sucralose, citric acid, BHT
Octenisept Oral Mono^®^ (Schülke & Mayr GmbH, Norderstedt, Germany)	Octenidine dihydrochloride (1000 µg/mL)	Glycerol, sodium gluconate, citric acid, disodium phosphate dihydrate, macrogolglycerol hydroxystearate, sucralose, water, mint flavor
Eludril Classic^®^ (Pierre Fabre, Castres, France)	Chlorhexidine digluconate (CHX; 1000 µg/mL)	Glycerin, alcohol, aqua, chlorobutanol, CI 16255, diethylhexyl sodium sulfosuccinate, flavor, limonene, menthol
Corsodyl^®^ (GlaxoSmithKline, Brentford, UK)	Chlorhexidine digluconate (2000 µg/mL)	Ethanol, macrogolglycerol hydroxystearate, sorbitol, peppermint oil, water
SeptOralMed^®^ (Avec Pharma, Wrocław, Poland)	Chlorhexidine digluconate (2000 µg/mL)	Aqua, glycerin, Peg 40 hydrogenated castor oil, limonene, eugenol, linalool, sodium saccharin
Perio Aid Intensive Care^®^ (Dentaid, Barcelona, Spain)	Chlorhexidine digluconate (1200 µg/mL), Cetylpyridinium chloride (CPC; 500 µg/mL)	Aqua, glycerin, propylene glycol, xylitol, PEG-40 hydrogenated castor oil, potassium acesulfame, sodium saccharin, neohesperidin dichalcone, aroma, CI 42090
Gum Paroex^®^ (Sunstar, Etoy, Switzerland)	Chlorhexidine digluconate (1200 µg/mL), Cetylpyridinium chloride (500 µg/mL)	Aqua, glycerin, propylene glycol, PEG-40 hydrogenated castor oil, aroma, sodium citrate, sucralose, citric acid, CI 14720
ProntOral^®^ (B Braun, Melsungen, Germany)	Polyaminopropyl biguanide (Polyhexanide, PHMB; 1500 µg/mL)	Aroma, sodium cyclamate, surfactants, excipients
Fomukal^®^ (Vipharm, Ożarów Mazowiecki, Poland)	Benzalkonium chloride (BAC; 125 µg/mL)	Sodium phosphate dibasic, sodium phosphate monobasic, calcium chloride, sodium chloride, water
Listerine Total Care^®^ (Johnson & Johnson, New Brunswick, NJ, USA)	Sodium fluoride (220 µg/mL), Eucalyptol, Thymol, Menthol, Alcohol	Aqua, sorbitol, aroma, poloxamer 407, benzoic acid, zinc chloride, aroma, sodium saccharin, methyl salicylate, sodium benzoate, sucralose, propylene glycol, CI 16035, CI 42090
Elmex Sensitive Plus^®^ (Colgate Palmolive, New York, NY, USA)	Olaflur, Potassium fluoride, total fluorine (250 µg/mL)	Aqua, propylene glycol, PEG-40 hydrogenated castor oil, aroma, PVP/dimethylaminoethylmethacrylate polycarbamyl polyglycol ester, saccharin, hydroxyethylcellulose, potassium hydroxide, polyaminopropyl biguanide
Meridol Gum Protection^®^ (Colgate Palmolive, New York, NY, USA)	Olaflur, Stannous fluoride, total fluorine (250 µg/mL)	Aqua, xylitol, PVP, PEG-40 hydrogenated castor oil, aroma, sodium saccharin, CI 42051
Baikadent mint^®^ (Herbapol Wrocław, Wrocław, Poland)	*Scutellaria baicalensis* root extract (concentration data is confidential and unavailable)	Aqua, sorbitol, xylitol, glycerin, PEG-40 hydrogenated castor oil, sodium benzoate, aroma, sodium lauryl sulfate, sodium carbonate, citric acid
Dentosept^®^ (Phytopharm, Nowe Miasto nad Wartą, Poland)	Liquid complex extract (910 mg/mL)	A liquid extract *from sage leaf *(*Salviae folium*), *peppermint herb* (*Menthae piperitae herba*), thyme herb (*Thymi herb*), chamomile flower (*Matricariae flos*), oak bark (*Quercus cortex*), arnica herb (*Arnica herba*), calamus rhizome (*Calami rhizomate*), benzocaine
Glimbax^®^ (Angelini Pharma, Rome, Italy)	Diclofenac (740 µg/mL)	Choline solution 50%, sorbitol, sodium benzoate, disodium edetate, acesulfame potassium, peach flavor enhancer, mint flavor enhancer, cochineal red (E 124), water

## Data Availability

Data sharing is not applicable to this article.
